# Effects of integrative therapy with Du Meridian moxibustion, ear acupuncture, and alprazolam on cardiac function and neurotransmitter levels in patients with coronary heart disease and insomnia: An observational study

**DOI:** 10.1097/MD.0000000000039318

**Published:** 2024-08-23

**Authors:** Xiaoxia Huang, Rui Li, Shuai Zhang, Kang Liu, Lirong Shen, Ying Shi, Zongde Hu

**Affiliations:** a Shanghai Pudong New Area Hospital of Chinese Traditional Medicine/Non Drug Therapy Center, Shanghai, China; b Rehabilitation Department, Shanghai Pudong New Area Dongming Community Health Service Center, Shanghai, China; c Chinese Traditional Medicine Orthopedics and Traumatology Department, Shuguang Hospital, Shanghai University of Traditional Chinese Medicine, Shanghai, China.

**Keywords:** coronary heart disease, Du Meridian acupuncture, ear acupuncture, insomnia, melatonin, neurotransmitters, therapeutic efficacy

## Abstract

To explore the effects of Du Meridian moxibustion combined with ear acupuncture on clinical symptoms and serum neurotransmitters in patients with coronary heart disease and insomnia. This study is a retrospective study. From June 2021 to May 2023, 116 patients with coronary heart disease and insomnia treated at our hospital were selected as subjects. They were divided into 2 groups according to the treatment. The control group received treatment with alprazolam, while the experimental group received Du Meridian moxibustion combined with ear acupuncture in addition to alprazolam treatment. The efficacy of the 2 groups was compared, and the levels of cardiac function indicators, serum melatonin, leptin, and neurotransmitters were measured. The total effectiveness rate in the experimental group was 93.10% (with a cure rate of 36.21%, a significant improvement rate of 41.38%, and an effective rate of 15.52%), which was significantly higher than the 79.31% in the control group (with a cure rate of 24.14%, a significant improvement rate of 32.76%, and an effective rate of 22.41%) (*P* < .05). Both groups exhibited an increase in left ventricular ejection fraction, stroke volume, and cardiac output after treatment compared to before treatment. Additionally, left ventricular end-systolic diameter decreased after treatment compared to before treatment, but the cardiac function was compared between the 2 groups after treatment (*P* > .05). In both groups, serum melatonin and serotonin (5-HT) levels increased after treatment compared to before treatment, while serum leptin, dopamine, and glutamate levels decreased after treatment compared to before treatment. Furthermore, the experimental group had higher serum melatonin, 5-HT, and gamma-aminobutyric acid levels compared to the control group, and lower serum leptin, dopamine, and glutamate levels compared to the control group (*P* < .05). The serum traditional Chinese medicine syndrome score and Pittsburgh sleep quality index score of the 2 groups decreased after treatment, and the experimental group was lower than the conventional group (*P* < .05). The combination of Du Meridian acupuncture with ear acupuncture in the treatment of insomnia in coronary heart disease can regulate the expression of serum melatonin, leptin, and neurotransmitters, alleviate symptoms, and improve therapeutic efficacy.

## 1. Introduction

Coronary heart disease is a common type of cardiovascular disease often associated with insomnia. This is because patients with coronary heart disease often experience heightened nervous system activity and a state of mild inflammation, which can exacerbate negative emotions and lead to insomnia. Furthermore, insomnia can worsen the hormonal imbalance and metabolic disturbances in patients with coronary heart disease, thus creating a vicious cycle.^[1]^ Alprazolam belongs to the class of benzodiazepine sedative drugs, and it provides effective short-term sedation and hypnotic effects. However, long-term use can lead to addiction, tolerance, and adverse effects such as drowsiness, dizziness, and fatigue. Discontinuation of the medication may also result in withdrawal symptoms.^[2]^

In traditional Chinese medicine, coronary heart disease is summarized in the category of “chest obstruction.” The heart dominates the blood vessels, and the lack of heart Qi cannot transport blood, so that the obstruction of collaterals leads to chest obstruction. Insomnia belongs to the category of “sleeplessness.” The spleen is in charge of transportation and transformation, and the spleen deficiency is lack of source, which cannot support the mind and cause insomnia. Therefore, coronary heart disease insomnia is more common in heart and spleen deficiency syndrome.^[3]^ Du Meridian acupuncture and ear acupuncture are both common external therapeutic methods in traditional Chinese medicine for treating coronary heart disease with insomnia, and they can effectively improve symptoms.^[4]^ However, there is relatively limited research on their impact on the serum neurotransmitter levels in patients. This study aims to investigate the effects of Du Meridian acupuncture combined with ear acupuncture on clinical symptoms and serum neurotransmitter levels in patients with coronary heart disease and insomnia.

## 2. Materials and methods

### 2.1. General data

This study was a retrospective study approved by the Ethics Committee of Shanghai Pudong New Area Hospital of traditional Chinese medicine. The study selected 116 patients with coronary heart disease and insomnia who were admitted to our hospital from June 2021 to May 2023 as the study population. They were divided into 2 groups according to the treatment. Both groups received routine treatment for coronary heart disease. The control group received treatment with alprazolam, while the experimental group received Du Meridian moxibustion combined with ear acupuncture in addition to alprazolam treatment. Inclusion criteria: coronary heart disease reference “stable coronary heart disease diagnosis and treatment guidelines”^[5]^ in the standard, ≥1 coronary artery stenosis ≥ 50%; insomnia reference “Chinese adult insomnia diagnosis and treatment guidelines”^[6]^ in the standard, and in line with the “Chinese medicine new drug clinical research guiding principles (Trial)”^[7]^ center spleen deficiency type standard: to chest pain, palpitations, dreams easy to wake up, chills limb cold main symptoms; with fatigue, pale complexion, dizziness, sweating, less food, abdominal distension, forgetfulness as secondary symptoms; light tongue, thin fur, weak pulse; age 18 to 75 years old, Pittsburgh sleep quality index (PSQI) > 7 points; not taking the treatment of insomnia-related drugs or have discontinued the treatment of insomnia-related drugs for at least 4 weeks; the patient himself or his family signed the consent. Exclusion criteria: with blood or immune system diseases; long-term use of stability drugs; with severe arrhythmia, cardiac function III, IV, malignant tumor; liver and kidney dysfunction; acute myocardial infarction within 6 months or complete revascularization within 12 months; have a history of drug abuse. Comparing the baseline data of the 2 groups, it was found that the data were balanced (*P* > .05). See Table 1. Comparisons of the pretreatment PSQI scores for both groups are presented in Table 2.

**Table 1:** Comparison of baseline data.

Group	n	Age (yrs)	Duration of coronary heart disease (yrs)	Duration of insomnia (yrs)	Gender (example)		Concomitant disease		
					Male	Female	Hypertension	Diabetes	Hyperlipemia
Conventional group	58	64.25 ± 6.74	7.85 ± 2.13	3.25 ± 1.06	32 (55.17)	26 (44.83)	22 (37.93)	16 (27.59)	15 (25.86)
Experimental group	58	63.79 ± 7.03	7.93 ± 2.41	3.22 ± 1.24	30 (51.72)	28 (48.28)	18 (31.03)	17 (29.31)	18 (31.03)
χ^2^/*t*		0.360	0.189	0.140	0.139		0.611	0.042	0.381
*P*		.720	.850	.889	.710		.435	.837	.537

**Table 2:** Comparisons of the pretreatment Pittsburgh Sleep Quality Index (PSQI) scores.

PSQI	Conventional group (n = 58)	Experimental group (n = 58)	*t*	*P*
Sleep quality	2.01 ± 0.25	1.97 ± 0.33	0.736	.463
Sleep onset latency	2.07 ± 0.31	2.11 ± 0.41	−0.593	.555
Total sleep time	2.26 ± 0.56	2.13 ± 0.44	1.390	.167
Sleep efficiency	2.43 ± 0.12	2.38 ± 0.66	0.568	.571
Sleep disturbances	2.03 ± 0.77	2.06 ± 0.51	−0.247	.805
Hypnotic	1.96 ± 0.82	2.02 ± 0.69	−0.426	.671
Daytime dysfunction	1.82 ± 0.17	1.79 ± 0.20	0.870	.386

### 2.2. Methods

Two groups of patients were given standard treatment for coronary heart disease, with instructions to quit smoking, quit drinking, and follow a low-salt, low-fat diet. Enteric-coated Aspirin Capsules (Manufacturer: Bayer Healthcare Co., Ltd., Shanghai, China, Approval No. National Drug Approval J20171021, Specification: 0.1 g) – 0.1 g, once daily (qd). Metoprolol Tartrate Tablets (Manufacturer: Shijiazhuang Yiling Pharmaceutical Co., Ltd., Shanghai, China, Approval No. National Drug Approval H20065355, Specification: 25 mg) – 25 mg, once daily (qd). Atorvastatin Calcium Tablets (Manufacturer: Tianfang Pharmaceutical Co., Ltd., Henan, China, Approval No. National Drug Approval H20203378, Specification: 10 mg) – 20 mg, once daily (qd). When experiencing an angina attack, you should take sublingual nitroglycerin tablets (Manufacturer: Shandong Xinyi Pharmaceutical Co., Ltd., Shandong, China, Approval No. National Drug Approval H37021445, Specification: 0.5 mg) by placing them under the tongue. Take 0.5 mg at a time, and repeat the dose every 5 minutes if the symptoms persist until relief is achieved. If the symptoms do not improve after 15 minutes, it is advisable to seek medical attention promptly.

The conventional group is administered zolpidem (Manufacturer: Shanghai Xinyi Pharmaceutical Co., Ltd., Approval No. National Drug Approval H31021282, Specification: 0.4 mg) at a dose of 0.4 mg, to be taken orally once daily (qd) before bedtime.

The experimental group receives combined treatment of Du Meridian moxibustion and auricular acupuncture in addition to zolpidem. Du Meridian Moxibustion: The patient is placed in a prone position with their back exposed. Du Meridian acupoints are selected for moxibustion, starting from the Da Zhui acupoint and extending down to the Yao Yangguan acupoint. Moxa sticks are ignited and placed in the moxibustion holes of a moxibustion box, which is then positioned over the Du Meridian acupoints. Moxibustion is administered for 20 minutes per session, with treatments given every other day. Ear Acupuncture: Specific ear acupoints, such as Shen Men, Subcortex, Occiput, and Heart, are selected. One side of the ear is sterilized conventionally, and a probe is used to locate the selected acupoints and their surrounding areas, identifying the reaction points. A single-use, sterile, 0.22 mm × 1.50 mm disposable acupuncture needle is inserted directly. The thumb and index finger of one hand are used to stabilize the auricle, while the middle finger supports the needle insertion at the back of the ear. The other hand uses 3 fingers to hold the needle, aiming at the auricular point, employing an oblique insertion technique. The depth of insertion is based on the thickness of the auricle. Upon needle insertion, the sensation of the needle was achieved and secured with adhesive tape, leaving the needle in place for 30 minutes. Upon removal, the puncture site was disinfected and compressed with a cotton ball to prevent bleeding. Unilateral acupuncture was alternated between both ears, conducted 5 times per week, and continued for 1 month. Patients are instructed to self-administer pressure on the acupuncture points 3 times a day, alternating between the left and right ears.

The efficacy of the 2 groups was evaluated after 4 weeks of continuous treatment. At 12 weeks, we reevaluated and compared the insomnia conditions of both patient groups to assess the long-term therapeutic efficacy.

### 2.3. Observed indexes

#### 2.3.1. Treatment efficacy criteria

Based on PSQI score reduction rate and insomnia symptoms. Cure: Symptoms completely disappear, and the PSQI score reduction rate is ≥95%. Significant Improvement: Symptoms significantly alleviate, with a PSQI score reduction rate between 70% and 95% (excluding 70%). Effective: Symptoms show relief, with a PSQI score reduction rate between 30% and 70% (excluding 30%). Ineffective: Symptoms do not improve or even worsen, with a PSQI score reduction rate <30%, or an increase in PSQI score.

#### 2.3.2. Cardiac function

Both groups underwent cardiac function evaluation using echocardiography before treatment and after 4 weeks of treatment. Cardiac function parameters measured included left ventricular ejection fraction (LVEF), stroke volume (SV), cardiac output (CO), and left ventricular end-systolic diameter (LVESD). The instrument used was the LOGIQ E11 or LOGIQ E10s color Doppler ultrasound machine from the United States.

#### 2.3.3. Testing method

Both groups collected 5 mL of venous blood samples in the morning on an empty stomach before treatment and after 4 weeks of treatment. The blood samples were placed in EDTA anticoagulant tubes and centrifuged at 4 °C and 3000 r/min for 10 minutes. The serum was separated and stored for further use. ELISA (Enzyme-Linked Immunosorbent Assay) was used to measure the levels of melatonin, leptin, as well as the neurotransmitters serotonin (5-HT), gamma-aminobutyric acid (GABA), dopamine (DA), and glutamate. The reagent kits were provided by Shanghai Tongwei Biotechnology Co., Ltd., and the instrument used was the Shimadzu MK3 microplate reader.

#### 2.3.4. Scoring criteria

Traditional Chinese medicine (TCM) syndrome score: Divided into primary symptoms (4 items) and secondary symptoms (6 items). Primary symptoms include chest discomfort, palpitations, frequent dreams, and easy awakening, aversion to cold with cold limbs. Depending on the severity reported by the patient, they are graded as none, mild, moderate, or severe, corresponding to 0, 2, 4, or 6 points. Secondary symptoms include dizziness and vertigo, fatigue and weakness, forgetfulness, dull complexion, excessive sweating, reduced appetite with abdominal distension. Depending on the severity reported by the patient, they are graded as none, mild, moderate, or severe, corresponding to 0, 1, 2, or 3 points. (2) PSQI score^[8]^: Consists of 7 dimensions with a total of 21 points. A total score >7 indicates the presence of insomnia, with a higher score indicating more severe insomnia.

### 2.4. Statistical processing

In this study, Excel table was used to collect and collate data, and SPSS25.0 was used for processing. The measurement data were expressed as ($\bar{\chi }\;\pm \;s$), and compared by *t* test. The enumeration data were described by use cases, and compared by χ^2^ test. *P* < .05 indicated that there was a statistical difference.

## 3. Results

### 3.1. Comparison of the curative effect

The total effective rate of the experimental group was 93.10% (cured 36.21%, markedly effective 41.38%, and effective 15.52%), which was higher than 79.31% (cured 24.14%, markedly effective 32.76%, and effective 22.41%) of the conventional group (*P* < .05). See Table 3.

**Table 3:** Comparison of the curative effect.

Group	n	Cure	Remarkable	Effective	In vain	Total effective rate
Conventional group	58	14 (24.14)	19 (32.76)	13 (22.41)	12 (20.69)	46 (79.31)
Experimental group	58	21 (36.21)	24 (41.38)	9 (15.52)	4 (6.90)	54 (93.10)
χ^2^						4.640
*P*						.031

### 3.2. Comparison of cardiac function

The cardiac function was compared (*P* > .05). The LVEF, SV, and CO of the 2 groups increased after treatment, and the LVESD decreased after treatment, but the cardiac function of the 2 groups was compared after treatment (*P* > .05). See Table 4.

**Table 4:** Comparison of cardiac function.

Group	n	LVEF (%)		SV (mL)		CO (L/min)		LVESD (mm)	
		Pretreatment	Posttreatment	Pretreatment	Posttreatment	Pretreatment	Posttreatment	Pretreatment	Posttreatment
Conventional group	58	56.86 ± 3.74	60.41 ± 4.22*	68.12 ± 4.65	74.85 ± 5.12*	2.96 ± 0.75	4.62 ± 0.93*	44.52 ± 3.96	38.52 ± 3.45*
Experimental group	58	55.45 ± 4.03	60.58 ± 4.75*	67.78 ± 5.02	75.02 ± 4.98*	2.91 ± 0.82	4.67 ± 0.88*	44.18 ± 4.52	38.27 ± 4.03*
*t*		1.953	0.204	0.378	0.181	0.343	0.297	0.431	0.359
*P*		.053	.839	.706	.856	.732	.767	.667	.720

Comparison before and after treatment.

* *P* < .05.

### 3.3. Comparison of serum melatonin and leptin

Serum melatonin and leptin were compared (*P* > .05). The serum melatonin in the 2 groups increased after treatment, and the serum leptin decreased after treatment. The serum melatonin in the experimental group was higher than that in the conventional group, and the serum leptin was lower than that in the conventional group (*P* < .05). See Table 5.

**Table 5:** Comparison of serum melatonin and leptin.

Group	n	Melatonin (pg/mL)		Leptin (µg/L)	
		Pretreatment	Posttreatment	Pretreatment	Posttreatment
Conventional group	58	40.52 ± 8.52	44.47 ± 8.63*	8.89 ± 2.12	6.95 ± 1.47*
Experimental group	58	39.89 ± 9.41	56.89 ± 10.17*	9.01 ± 2.05	5.23 ± 1.21*
*t*		0.378	7.092	0.310	6.880
*P*		.706	.000	.757	.000

Comparison before and after treatment.

* *P* < .05.

### 3.4. Comparison of serum neurotransmitters

Serum neurotransmitters were compared (*P* > .05). The levels of serum 5-HT and γ-aminobutyric acid in the 2 groups were higher than those before treatment, and the levels of serum DA and glutamic acid were lower than those before treatment. The levels of serum 5-HT and γ-aminobutyric acid in the experimental group were higher than those in the conventional group, and the levels of serum DA and glutamic acid were lower than those in the conventional group (*P* < .05). See Table 6.

**Table 6:** Comparison of serum neurotransmitters.

Group	n	5-HT (ng/mL)		DA (ng/L)		Glutamic acid (mg/L)		Gamma-aminobutyric acid (mg/L)	
		Pretreatment	Posttreatment	Pretreatment	Posttreatment	Pretreatment	Posttreatment	Pretreatment	Posttreatment
Conventional group	58	10.12 ± 2.16	13.78 ± 2.52*	658.52 ± 84.63	459.63 ± 72.36*	323.32 ± 48.95	267.03 ± 35.52*	221.41 ± 56.96	271.02 ± 60.74*
Experimental group	58	9.98 ± 2.24	16.47 ± 2.66*	649.78 ± 88.25	374.52 ± 61.12*	319.74 ± 52.27	225.02 ± 30.41*	209.87 ± 60.44	307.52 ± 67.83*
*t*		0.343	5.591	0.544	6.843	0.381	6.842	1.058	3.053
*P*		.733	.000	.587	.000	.704	.000	.292	.003

Comparison before and after treatment.

* *P* < .05.

### 3.5. Comparison of TCM syndrome score and PSQI score

The TCM syndrome score and PSQI score were compared (*P* > .05). The serum TCM syndrome score and PSQI score of the 2 groups decreased after treatment, and the experimental group was lower than the conventional group (*P* < .05). See Table 7.

**Table 7:** Comparison of TCM syndrome score and PSQI score.

Group	n	Traditional Chinese medicine syndrome points (score)		PSQI score (score)	
		Pretreatment	Posttreatment	Pretreatment	Posttreatment
Conventional group	58	29.78 ± 2.57	14.86 ± 1.96*	14.85 ± 2.12	7.83 ± 1.06*
Experimental group	58	30.01 ± 2.71	9.78 ± 1.24*	14.93 ± 2.07	6.21 ± 0.67*
t		0.469	16.681	0.206	9.839
*P*		.640	.000	.837	.000

Comparison before and after treatment.

* *P* < .05.

### 3.6. Comparison of long-term PSQI scores

Finally, we compared the scores for each item of the PSQI 3 months posttreatment between the 2 patient groups to observe the relative long-term therapeutic effects, as shown in Table 8. The results indicated that only the “Sleep Quality” item showed a significant difference between the groups, with the experimental group scoring lower at 1.97 ± 0.41 compared to the conventional group at 2.11 ± 0.34. Although there was a trend towards lower scores in the experimental group for the other items, these differences were not statistically significant.

**Table 8:** Comparisons of the long-term Pittsburgh Sleep Quality Index (PSQI) scores.

PSQI	Conventional group (n = 58)	Experimental group (n = 58)	*t*	*P*
Sleep quality	2.11 ± 0.34	1.97 ± 0.41	2.002	**.048**
Sleep onset latency	1.86 ± 0.68	1.82 ± 0.46	0.371	.711
Total sleep time	2.03 ± 0.36	1.98 ± 0.55	0.579	.564
Sleep efficiency	2.17 ± 0.22	2.08 ± 0.42	1.446	.151
Sleep disturbances	1.95 ± 0.62	1.96 ± 0.51	−0.095	.925
Hypnotic	1.90 ± 0.31	1.87 ± 0.20	0.619	.537
Daytime dysfunction	1.70 ± 0.11	1.69 ± 0.20	0.334	.739

## 4. Discussion

Coronary heart disease accompanied by insomnia is considered a dual heart condition. Patients with coronary heart disease may experience discomfort such as palpitations and chest pain, which can lead to insomnia and anxiety. Insomnia, in turn, can increase sympathetic nervous system activity, leading to elevated blood pressure, increased heart rate, and exacerbation of myocardial ischemia.^[9]^ Some studies suggest that chronic insomnia is an independent risk factor for worsening coronary heart disease.^[1]^ Currently, in Western medicine clinical practice, sedative-hypnotic drugs are generally the first choice for intervention in insomnia associated with coronary heart disease. However, the overall efficacy is not always very satisfactory. An increasing number of scholars believe that incorporating traditional Chinese medicine as adjunctive therapy can improve treatment outcomes. Du Meridian moxibustion is a special type of moxibustion therapy, where the Du Meridian is considered the “sea of yang meridians.” Moxibustion on this meridian can help to warm and nourish the organs, as well as harmonize Qi and blood. Ear acupuncture is a special type of acupuncture therapy. Traditional Chinese medicine believes that the ear contains numerous reflex points corresponding to internal organs. By stimulating these ear acupoints, it is believed that one can regulate the balance of Qi and blood, harmonizing Yin and Yang within the body.^[10]^

This study found that compared to the conventional group, the experimental group had a higher overall effective rate. In both groups, the serum traditional Chinese medicine syndrome scores and PSQI scores decreased after treatment, with the experimental group showing lower scores compared to the conventional group. This result suggests that the combined treatment of Du Meridian moxibustion and ear acupuncture for insomnia in patients with coronary heart disease can alleviate TCM symptoms, improve treatment efficacy, and enhance sleep quality. This is likely due to the wide application area and strong heat generated by Du Meridian moxibustion. During moxibustion, the burning moxa stick can transmit a warming effect to the back, dilate local blood vessels, improve microcirculation, and enhance myocardial blood perfusion, thereby alleviating a range of symptoms caused by myocardial ischemia.^[11]^ The ear area contains the distribution of the vagus nerve, and the auricular branch of the vagus nerve can establish direct connections with various brain regions, including the solitary tract nucleus, locus coeruleus, amygdala, hippocampus, and others. By stimulating ear acupoints, it is believed that the activation of a bidirectional reflex pathway between the internal organs, central nervous system, and the ear can improve cardiac function and regulate neural excitability.^[12]^

Coronary heart disease patients often exhibit a gradual decline in cardiac function, which can ultimately lead to heart failure or even death.^[13]^ In this study, echocardiography was used to measure cardiac function indicators before and after treatment in both groups. The results showed that in both groups, LVEF, SV, and CO increased after treatment, while LVESD decreased. However, when comparing the posttreatment cardiac function between the 2 groups, there was no statistically significant difference. This result suggested that both treatment approaches contribute to the protection of patients’ cardiac function, but the combined treatment of Du Meridian moxibustion and ear acupuncture did not show a significant advantage in improving cardiac function. This may be due to the relatively short observation period of only 4 weeks in this study, while the improvement of cardiac function is a long-term process. Future research should continue to strengthen follow-up and further investigate whether the combined treatment of Du Meridian moxibustion and ear acupuncture demonstrates more significant advantages in improving patients’ cardiac function in the long term.

Melatonin is a hormone secreted by the pineal gland in the brain, and its secretion follows a circadian rhythm, regulating the sleep–wake cycle and improving sleep quality. Leptin is a hormone secreted by adipose tissue, with its secretion peaking at night. Sleep disorders can disrupt the secretion of leptin.^[14]^ The neurotransmitters 5-HT and γ-aminobutyric acid are inhibitory neurotransmitters, which can block the excitatory conduction of nerves and regulate the periodic rhythm of sleep and promote sleep. DA and glutamate are excitatory neurotransmitters with physiological functions such as excitation and arousal.^[15,16]^

This study found that in both groups, serum melatonin, 5-HT, and gamma-aminobutyric acid (GABA) levels increased after treatment, while serum leptin, DA, and glutamate levels decreased. Additionally, the experimental group had higher serum levels of melatonin, 5-HT, and GABA compared to the conventional group, while their serum levels of leptin, DA, and glutamate were lower. This result suggests that the combined treatment of Du Meridian moxibustion and ear acupuncture for insomnia in patients with coronary heart disease can regulate the expression of serum melatonin, leptin, and neurotransmitters. This is one of the important mechanisms behind its effectiveness in treating insomnia associated with coronary heart disease. Stimulation of ear acupuncture can improve blood flow in the vertebral-basilar artery, increase the supply of blood and oxygen to the central nervous system and the heart, and regulate the secretion of brain melatonin as well as excitatory and inhibitory neurotransmitters. This, in turn, helps regulate the function of brain regions related to the sleep–wake cycle.^[17]^

Finally, we included in our article the insomnia conditions of both patient groups 3 months after treatment. While there were differences between the groups, they were minimal. We believe this is reasonable as the treatment in this study was experimental and targeted at a specific phase of intervention. Achieving long-term efficacy in controlling insomnia would require regular cyclic treatment. However, these results provide a favorable indication of the long-term effectiveness of the treatment.

In summary, the combined treatment of Du Meridian moxibustion and ear acupuncture for insomnia in patients with coronary heart disease can regulate the expression of serum melatonin, leptin, and neurotransmitters. This approach helps alleviate symptoms and improve treatment efficacy.

## Author contributions

**Conceptualization:** Xiaoxia Huang, Rui Li, Shuai Zhang, Kang Liu, Lirong Shen, Ying Shi, Zongde Hu.

**Data curation:** Xiaoxia Huang, Rui Li, Shuai Zhang, Kang Liu, Lirong Shen, Ying Shi, Zongde Hu.

**Formal analysis:** Xiaoxia Huang, Rui Li, Shuai Zhang, Kang Liu, Lirong Shen, Zongde Hu.

**Funding acquisition:** Xiaoxia Huang, Kang Liu, Lirong Shen, Zongde Hu.

**Investigation:** Xiaoxia Huang, Kang Liu, Lirong Shen.

**Supervision:** Rui Li, Ying Shi, Zongde Hu.

**Writing – original draft:** Rui Li, Shuai Zhang.

**Writing – review & editing:** Rui Li, Shuai Zhang.

**Methodology:** Kang Liu.
